# Supplementary material for: A Systematic Review of Dynamic Forces and Kinematic Indicators of Front and Roundhouse Kicks across Varied Conditions and Participant Experience
Source: Sports (Basel). 2023 Jul 28;11(8):141. doi: 10.3390/sports11080141 (PMC10459763; doi:10.3390/sports11080141)
Supplement: Supplementary file 1 [file sports-11-00141-s001.zip › Supplementary materials.pdf]

Table S1. The search terms used in the review to identify dynamics forces and kinematic indicators of the front and roundhouse kick. These search terms were used in Web of Science, PubMed, and SportDicsus databases. Full text, search was conducted from 1982 to 19th May 2022, Humans, English.

Web of Science	Topic ((((((((ALL=(front kick*)) OR ALL=(roundhouse kick*)) OR ALL=(Mae-Geri*)) OR ALL=(Apchagi*)) OR ALL=(Dollyo*)) OR ALL=(Mawashi*)) NOT ALL=(Swimming*)) NOT ALL=(Football*)) NOT ALL=(Rugby*)) NOT ALL=(Futsal*)<br>Limit to: Date from 1982 to 19th May 2022, Article, English
PubMed	(((((((((front kick*[Title/Abstract])) OR (roundhouse kick*[Title/Abstract]))) OR (Mae-Geri*[Title/Abstract]))) OR (Apchagi*[Title/Abstract]))) OR (Dollyo*[Title/Abstract]))) OR (Mawashi*[Title/Abstract]))) NOT (Swimming*[Title/Abstract]))) NOT (Footbal*[Title/Abstract]))) NOT (Rugby*[Title/Abstract]))) NOT (Futsal*[Title/Abstract]))<br>Limit to: from 1982 to 19th May 2022, Article, Humans, English
SportDiscus	TITLE-ABS-KEY front kick* OR roundhouse kick* OR Mae-Geri* OR Apchagi* OR Dollyo* OR Mawashi* NOT Swimming* NOT Football* NOT Rugby* NOT Futsal*<br>Limit to: from 1982 to 19th May 2022, Article, English

Table S2. Summary of studies

**Authors**	**Participants**	**Kick Type**	**Measure equipment**	**Variables**
Abraham et al., 2001	8 elite men	Front and roundhous e kick Martial arts	Peak Motus system (Peak Performance Technologies, USA, 200 Hz)	Maximum velocity (foot)
Branco et al., 2019	9 elite men, 54.2 ± 3.9 y<br>173 ± 6.6 cm, 78.5 ± 7.5 kg<br>24 elite men, 23 ± 5.8 y, 174.2 ± 7.9 c, 73.1 ± 14.6 kg	Front kick Karate	High-speed camera (Casio EX-FH20, sampling frequency of 210 Hz)	Maximum velocity (foot, knee, hip)<br>Maximum angular velocity<br>Initial, contact, max., and min. angle
Cynarski et al., 2018	One elite man, 36 y, 97 kg, 177 cm	Front kick Karate	Force plate (Kistler, type 9286AA, Kistler, Switzerland).	Execution time
Detjareny et al., 2012	5 sub-elite men, 21.4 ± 2.3 y,<br>169.2 ± 6.6 cm, 60.6 ± 10.4 kg	Roundhous e kick Taekwond o	Eight 100-Hz cameras (Natural Point Inc., USA), DAQ NI USB–6008 (National Instruments, USA)	Maximum velocity (foot)<br>Maximum angular velocity<br>Response time
Diniz et al., 2018	17 elite men, 23.7 ± 1.2 y, 174 ± 2 cm, 73.8 ± 2.5 kg<br>15 elite men, 25.2 ± 0.8 y, 178 ± 1.43 cm, 71.3 ± 3.6 kg<br>15 elite men, 28 ± 1.3y, 175 ± 1.74 cm, 74.8 ± 2.6 kg	Roundhous e kick Taekwond o, Karate, Muaythay	VICON Motion Capture System, 6 cameras, 200Hhz)	Angular Velocity

Dworak et al., 2003	21 sub-elite men, 26.4 ± 6.8 y, 179 ± 5.2 cm, 78.5 ± 6.5 kg	Front kick Karate	Force plate (Kistler 9261A)<br>MRC (device for measuring velocity)	Impact Force<br>Maximum velocity (foot)
Estevan et al., 2011	13 elite and 14 sub-elite men, 26.6 ± 2.2 y, 177 ± 8.9 cm, 72 ± 12.7 kg	Roundhouse kick Taekwondo	Force plate (A201 model by Tekscan Company Inc., South Boston, MA, USA)	Impact time<br>Execution time (during the hit)<br>Impact time (during the hit)
Estevan et al., 2012	15 sub-elite men, 25 ± 5.7 y, 73.4 ± 3.7 kg	Roundhouse kick Taekwondo	Force plate (A201 model by Tekscan Company Inc., South Boston, MA, USA)	Impact Force
Estevan & Falco, 2013	12 elite men, 24.3 ± 3y, 179 ± 6 cm, 77.9 ± 10.3 kg<br>21 sub-elite men, 25.7 ± 7 y, 178 ± 9 cm, 75.7 ± 11.8 kg	Roundhouse kick Taekwondo	The dummy (706 FSD ®, USI Universal Company, India)	Impact Force<br>Execution time
Estevan et al., 2014	33 sub-elite men, 24.4 ± 5.4y, 175 ± 0,1 cm, 72.5 ± 13.36 kg	Roundhouse kick Taekwondo	Force plate (A201 model by Tekscan Company Inc., South Boston, MA, USA)	Impact Force<br>Execution time
Falco et al., 2009	15 elite men, 23.5 ± 3.3y, 174 ± 0.12 cm, 69.97 ± 13.76 kg<br>16 novice men, 23.5 ± 3.3 y, 172 ± 0,1 cm, 68.12 ± 13 kg	Roundhouse kick Taekwondo	Five piezo-resistant pressure sensors (A201 model by FLEXIFORCE Company)	Impact Force<br>Execution time
Falco et al., 2013	13 elite men, 21.57 ± 4.75 y, 179 ± 0.1 cm, 76.8 ± 10 kg<br>21 sub-elite men, 21.57 ± 4.11y, 176 ± 0,1 cm, 75.5 ± 11.2 kg	Roundhouse kick Taekwondo	Force plate (2000 Hz)	Impact Force<br>Execution time
Gavagan et al., 2017	8 elite men, 22.3 ± 4.1 y, 174.6 ± 0.1 cm, 65.6 ± 8.4 kg<br>8 elite men, 28.6 ± 9.5 y, 178 ± 0.05 cm, 95.8 ± 13.4 kg<br>8 elite men, 30.3 ± 10.7 y, 179 ± 0.14 cm, 4.5 ± 20.1 kg	Roundhouse kick Muay Thai Taekwondo Karate	Qualisys Motion Capture System (Qualisys AB, Gothenburg, Sweden) 500 Hz<br>PowerLab system (PowerLab 8SP, ADInstuments, Inc. USA) 1000 Hz	Impact Force<br>Maximum velocity (foot)<br>Maximum angular velocity
Goethel et al., 2019	7 elite men, 26.3 ± 6.9 y, 170 ± 10 cm, 77.5 ± 12.8 kg<br>7 sub-elite men, 27.5 ± 6.1 y, 170 ± 10 cm, 75.1 ± 8.9 kg	Front kick Karate	Vicon Nexus (7 cameras, sampling frequency 250 f/s)	Maximum velocity (foot)<br>Maximum angular velocity<br>Continuous relative phase
Górski & Orysiak, 2019	6 elite men, 20 ± 3.2 y, 185 ± 8.5 cm, 75.3 ± 10.9 kg	Roundhouse kick Taekwondo	Two tri-axial accelerometers with triaxial gyroscope modules, 500 Hz	Impact Force
Chinnasee et al., 2017	6 elite men, 21.5 ± 1.09 y, 172.6 ± 4.94 cm, 87.45 ± 5.93 kg	Roundhouse kick Taekwondo	Vicon T10s (Oxford Metrics, UK) 100 Hz	Maximum Velocity (foot)

Jeon et al. 2021	11 elite men, 21.27 ±1.19 y, 72.5 ± 9.96 kg<br>11 novice men, 22.91 ± 1.62 y, 170 ± 7.99 cm, 75.2 ±10.49 kg	Roundhouse kick Taekwondo	Prime 13 (Optitrack, USA, eight motion capture cameras)	Total execution time
Jung & Park, 2018	10 elite men, 21.7 ± 0.5 y, 173.1 ± 4.3 cm, 60.6 ± 3.7 kg	Roundhouse kick Taekwondo	MX-13 Vicon™ motion capture system (Vicon, Ltd., Oxford, UK) 250 Hz	Maximum velocity (foot)
Kim et al., 2011	12 elite men, 20.4 ± 8.4 y, 180 ± 0.04 cm, 71.9 ± 8.4 kg	Roundhouse kick Taekwondo	Six-camera motion analysis system (Hawk® Digi- tal Real Time System, Motion Analysis System, Santa Rosa, CA, USA) 200 Hz	Maximum velocity (foot)<br>Execution time
Liu et al., 2021	19 elite men, 19.9 ±0.98 y, 177.57 ± 6.07 cm, 67.84 ± 9.65 kg	Roundhouse kick Taekwondo	Eight-camera motion analysis system (Eagle, Motion Analysis Corp., Santa Rosa USA) 200 Hz	Maximum velocity (foot, knee, hip)<br>Maximum angular velocity
Moreira et al., 2018	5 elite men and 2 women, 23.6 ± 2.1 y, 168 ± 5 cm, 69 ± 9.5 kg<br>5 sub-elite men and 2 women, 22.4 ± 2.1 y, 174 ± 11 cm, 66.8 ± 14.2 kg	Roundhouse kick Taekwondo (Execution into the Air)	Force platform OR-6 (AMTI®)<br>NEXUS motion capture system (Vicon®, v.2.0) seven cameras 250 Hz	Maximum velocity (foot, knee, hip)<br>Maximum angular velocity<br>Execution time<br>Ground reaction forces
Moreira et al., 2021	5 elite men and 2 women, 23.6 ± 2.1 y, 168 ± 5 cm, 69 ± 9.5 kg<br>5 sub-elite men and 2 women, 22.4 ± 2.1 y, 174 ± 11 cm, 66.8 ± 14.2 kg	Roundhouse kick Taekwondo (Execution into the target)	Vicon1MX13 cameras, sampled at 250 Hz	Maximum velocity (foot, knee, hip)<br>Maximum angular velocity<br>Execution time
Nien et al., 2007	6 elite men	Roundhouse kick Taekwondo	Eight high speed cameras (sampling rate 120 Hz)	Maximum Velocity (foot and knee)<br>Maximum angular velocity
Olsen et al., 2003	18 novice men, 27 ± 7.5 y, 181.5 ± 10.5 cm, 85 ± 11 kg	Front kick Martial arts	Force plate (1000 Hz)	Impact Force
Petre & Teodoru, 2013	10 sub-elite men, 21 – 23 y	Roundhouse kick Taekwondo	Panasonic cameras, Video captures with Quintic sports video analysis system	Maximum velocity (foot)
Portela et al., 2014	8 elite men, 30 ± 10.2 y, 178.9 ± 4.9 cm, 85.3 ± 12.2 kg	Front kick Karate	Casio FH25, with a frequency of 240 Hz	Maximum velocity (foot)
Pozo et al., 2011	8 elite men, 24.2 ± 10.6 y, 174.4 ± 8.6 cm, 70.3 ± 9.3 kg<br>9 sub-elite men, 37.9 ± 9 y, 176.4 ± 7.9 cm, 76 ± 11.3 kg	Front kick Karate	2D high-speed camera (Basler piA640-210gc, Basler AG)<br>3D-Force Plate (Arsalis, Louvain-la-Neuve, Belgium)	Total and phase execution time<br>Impact force

Quinzi et al., 2013	6 elite men, 24.2 ± 10.6 y, 174.4 ± 8.6 cm, 70.3 ± 9.3 kg<br>6 sub-elite men, 37.9 ± 9 y, 176.4 ± 7.9 cm, 76 ± 11.3 kg	Roundhouse kick Karate	Vicon System, Oxford Metrix, UK, 120 Hz	Maximal angular Velocity<br>Execution time
Quinzi et al., 2016	6 elite men, 15.5 ± 1 y, 170 ± 10 cm, 57.5 ± 4.8 kg	Roundhouse kick Karate	Vicon System, Oxford Metrix, UK, 100 Hz	Maximal angular Velocity<br>Execution time
Sbriccoli et al., 2010	6 elite men, 24.8 ± 1 y, 73.8 ± 4 kg<br>6 novice 27.8 ± 1 y, 77 ± 3.6 kg	Front kick Karate	MARG (MTx, Xsens Motion Technologies, 3D linear accelerometer, 3D angular rate sensor, 3D magnetometer)	Angular Velocity
Tang et al., 2007	6 elite men	Roundhouse kick Taekwondo	10 high speed cameras (Eagle cameras, Motion Analysis Corporation, Santa Rosa, CA, USA) 120 Hz	Maximum velocity (foot)<br>Angular velocity (hip, knee)
Thibordee & Prasartwuth, 2014	8 elite men, 24.3 ± 5.9 y, 172 ± 0.05 cm, 88.7 ± 4.2 kg<br>8 sub-elite men, 16.8 ± 7.7 y, 162 ± 10 cm, 49.3 ± 12.9 kg	Roundhouse kick Taekwondo	Mono-axial force transducer (2 kN; LC 1205-K200, A&D Co Ltd., Japan, 200 Hz).	Impact force
Vagner et al., 2018a	6 novice men, 22.2 ± 1.5 y, 180.6 ± 4.8 cm, 78.8 ± 5.8 kg	Front kick Musado	Triaxial force plate Kistler 9281 (1000 Hz)	Maximum force<br>Impulse<br>Time to reach maximum force
Vagner et al., 2018b	5 sub-elite men, 23.3 ± 1.7 y, 175.5 ± 4.5 cm, 74.3 ± 6.2 kg	Front kick Musado	Triaxial force plate Kistler 9281 (1000 Hz)	Maximum force<br>Impact force<br>Time to reach maximum force
Vagner et al., 2020	25 sub-elite men, 27.7 ±7.2 y, 180.5 ± 6.5 cm, 83.8 ± 6.1 kg	Front kick Musado	Triaxial force plate Kistler 9281 (2000 Hz)<br>Qualisys 2.2, Sweden (500 Hz).	Maximum force<br>Impact Force<br>Impulse<br>Maximum Velocity
Vagner et al., 2021	8 sub-elite men, 21.3 ± 0.9 y, 179.6 ± 5.2 cm, 76.8 ± 6.4 kg<br>8 sub-elite men, 22 ± 1 y, 184 ± 6.5 cm, 82.1 ± 5.4 kg	Front kick Musado	Triaxial force plate Kistler 9281 (1000 Hz)<br>Qualisys 2.2, Sweden (1000 Hz).	Maximum and Impact Force<br>Impulse<br>Maximum Velocity<br>Angular Velocity<br>Execution time
Vagner et al., 2022a	12 elite men, 31.8 ± 7.4 y, 179.8 ± 5.4 cm, 81.7 ± 6.1 kg<br>12 sub-elite men, 22.5 ± 2 y, 182.4 ± 6.3 cm, 81.7 ± 6.1 kg	Front kick Musado	Triaxial force plate Kistler 9281 (1000 Hz)<br>Qualisys 2.2, Sweden (200 Hz).	Maximum and Impact Force<br>Impulse<br>Maximum Velocity<br>Angular Velocity<br>Execution time
Vencesbrito et al., 2014	14 elite men, 24 ± 7 y, 171 ± 7 cm, 72 ± 19 kg<br>16 novice men, 23 ± 6 y, 179 ± 6 cm, 73 ± 10 kg	Front kick Karate	High speed camera Casio EX-FH20 (sampling frequency of 210 Hz)	Maximum Velocity<br>Contact time, onset time<br>Max. and min. angle
Wasik et al. 2015	6 sub-elite men, 16.5 ± 1 y, 176.5 ± 7.5 cm, 64.14 ±10.9 kg	Front kick Taekwondo	Italian system (Smart-D, made by BTS S.p.A.; six cameras, 120 Hz)	Total execution time<br>Maximum velocity of the foot and knee

Wasik & Góra. 2016	One elite man, 28 y, 172 cm, 68 kg	Front kick Taekwondo	10 NIR Vicon MX-T40 cameras, speed of 370 frames per/s, 4 megapixels (2352 x 1728 px) in 10-bit greyscale	Maximum velocity of the foot<br>Execution kick into: Air, Table tennis ball, Shield
Wasik et al. 2018	8 sub-elite men, 18.3 ± 1.7 y, 176.2 ± 3 cm, 70.4 ± 6 kg	Front kick Taekwondo	VICON Motion Systems MX40, Oxford Metrics Ltd., Oxford, England, 250 frames/s)	Maximum velocity (foot)<br>Execution time into: Air, Board, Small-ball, Shield
Wasik et al. 2021	15 elite men, 22.5 ± 6.2 y, 175.7 ± 8.4 cm, 71.9 ± 11.5 kg	Roundhouse kick Taekwondo	10 NIR Vicon MX-T40, resolution of 4 MP (2352 x 1728 pixels) 370 Hz	Maximum velocity (foot, knee, hip)

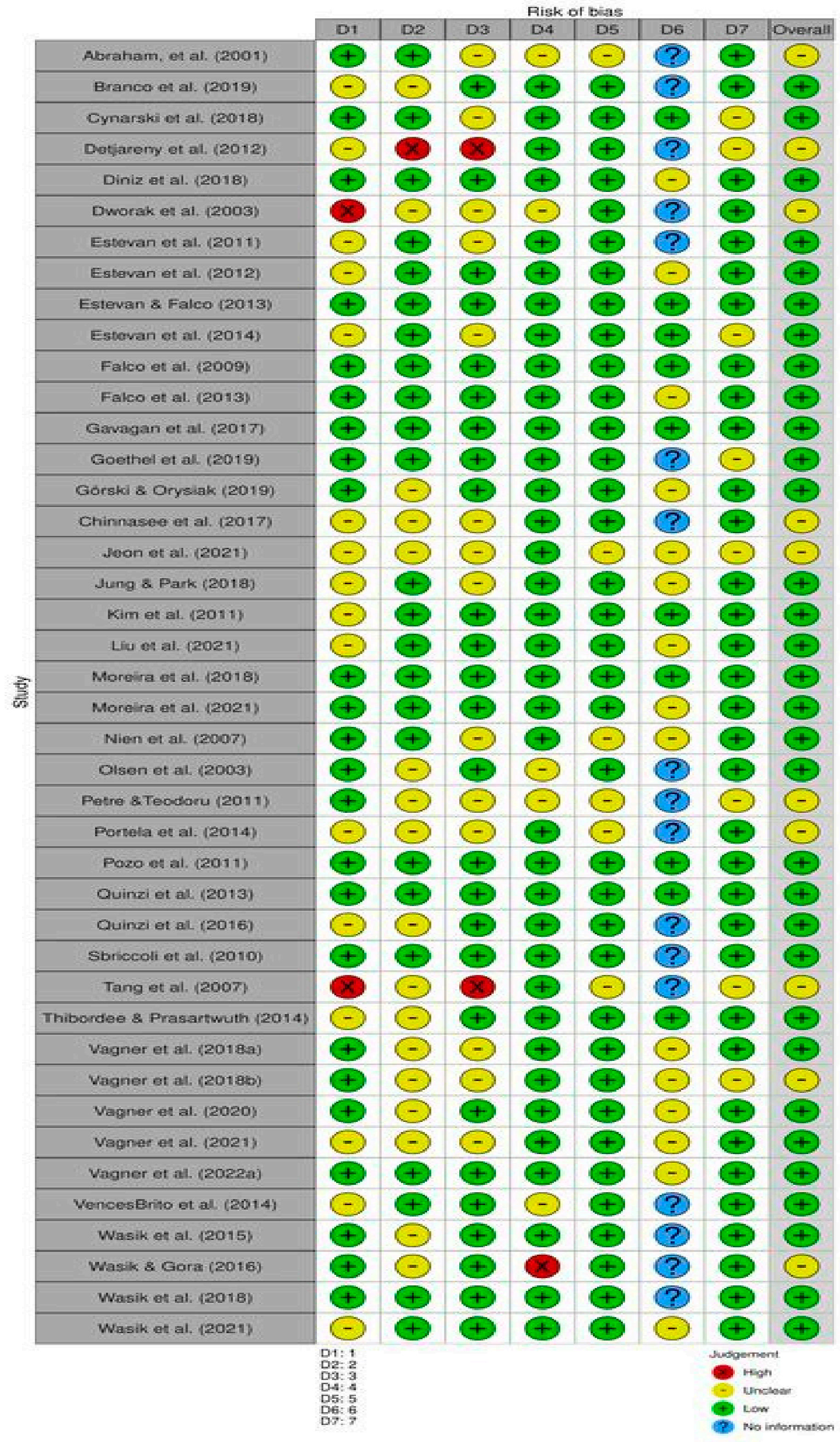

Figure S1. Assessment of methodological quality and risk of bias

Note: low risk of bias "+", some concerns "–", high risk of bias "x", and no information "?"; D1 – Was an informative and balanced summary of what was done and what was found provided in the abstract? D2 – Was the scientific background of the study explained? D3 – Were the eligibility criteria and the sources and methods of selecting participants stated? D4 – Was the condition measured in a standard, reliable way for all participants included in the study? D5 – Was the execution of the measurement of the dynamic or kinematic indicators described in sufficient detail to permit its replication? D6 – Were any efforts to address potential sources of bias described? D7 – Were outcomes and conclusions clearly defined?
